# Comprehensive analysis of the circRNA expression profile and circRNA-miRNA-mRNA network in pelvic organ prolapse

**DOI:** 10.3389/fgene.2024.1527223

**Published:** 2025-01-20

**Authors:** Qian Wang, Zuoxi He, Lisha Ding, Yuqing Liu, Xiaoli Zhang, Tao Wang, Xiaoyu Niu

**Affiliations:** ^1^ Laboratory of Metabolomics and Gynecological Disease Research, Key Laboratory of Birth Defects and Related Diseases of Women and Children of Ministry of Education, West China Second University Hospital, Sichuan University, Chengdu, Sichuan, China; ^2^ Department of Obstetrics and Gynecology, Key Laboratory of Birth Defects and Related Diseases of Women and Children of Ministry of Education, West China Second University Hospital, Sichuan University, Chengdu, Sichuan, China

**Keywords:** pelvic organ prolapse, circRNA sequencing, peripheral blood, differentially expressed circRNAs, circRNA-miRNA-mRNA network

## Abstract

Pelvic organ prolapse (POP) is a common gynecological disease caused by pathological defects, lesions, or mechanical weakening of the support structures of the pelvic floor. However, its pathogenesis is unclear. Circular RNAs (circRNAs) are covalently closed, endogenous biomolecules, which are thought to play an important role on skeletal muscle development by regulating gene expression. In this study, five pairs of peripheral blood samples from control and POP groups were used for circRNA sequencing analysis to obtain differential expression profiles. A total of 75 differentially expressed circRNAs (DEcircRNAs) were identified (fold change >2.0, P < 0.05). Furthermore, RT-qPCR confirmed that the expression levels of two circRNAs (hsa_circ_0067962 and hsa_circ_0057051) were significantly lower in the POP group. The two validated DEcircRNAs were abundantly involved in the collagen catabolic process. The circRNA-miRNA-mRNA network of two DEcircRNAs comprised nine mRNAs, which indicated that hsa_circ_0067962 and hsa_circ_0057051 may be involved in the pathogenesis of POP by regulating these nine mRNAs.

## 1 Introduction

Pelvic organ prolapse is a common problem among women in which the uterus, bladder, and/or rectum protrude from the vagina. This may be due to pathological defects, lesions, or mechanical weakening of the support structures of the pelvic floor (Weintraub et al., 2020). The prevalence of POP is 3%–6% when defined by symptoms, and 41%–50% based on vaginal examination (Barber and Maher, 2013). Despite the high prevalence of POP, its pathophysiological course remains unclear. Epidemiological studies indicate that POP is a common disease with a multifactorial etiology, including genetic predisposition, age, vaginal delivery, vaginal parity, obesity, and chronic cough (American College of Obstetricians and Gynecologists and the American Urogynecologic Society, 2019). How these factors interact and influence the biopathological changes in the body, especially in the pelvic region, has long been researched. These risk factors are thought to damage structural components of female pelvic floor, including pelvic floor skeletal muscles, and therefore may result in POP (Alperin et al., 2016). Age-related changes in skeletal muscle architecture and pathological accumulation of collagen or fibrosis in the extracellular matrix of muscle tissue were observed in sarcopenia, which is a degenerative and generalized skeletal muscle disorder involving the loss of muscle mass and function (Wilkinson et al., 2021). While these muscle changes also occurred in the pelvic floor muscles and participated in the pathogenesis of POP, indicating that there may be a pathophysiological association between POP and skeletal muscle degenerative disorder (Silva et al., 2021). Hence, investigating the potential molecular mechanisms that contribute to POP pathogenesis is urgently needed.

Circular RNAs are a group of endogenous single-stranded RNA characterized by a covalently closed cyclic structure. Recent studies suggest that circRNAs have unique tissue and developmental stage-specific expression patterns and play crucial roles in several human chronic diseases, including diabetes mellitus, chronic inflammatory diseases, and cancer, which all have complicated multifactorial etiology (Fang et al., 2018). An increasing number of studies have revealed that circRNAs play an important role on skeletal muscle development by regulating gene expression. For instance, circLMO7 promotes myoblast proliferation and inhibits myoblast differentiation, circFUT10 and circSNX29 inhibit proliferation and promote differentiation of myoblasts (Legnini et al., 2017; Yue et al., 2019). However, the role of circRNAs in POP remains poorly understood. According to the hypothesis of competitive endogenous RNA (ceRNA), circRNAs regulate the expression of downstream target mRNA by sponging miRNAs (Zhang et al., 2021). The ceRNA-based regulatory network, that is, the circRNA-miRNA-mRNA axis, has been identified in various diseases (Mohanapriya et al., 2022).

In this study, we identified the differential expression profiles of circRNAs in the peripheral blood of POP and control groups using circRNA sequencing analysis. A series of bioinformatics analyses was performed to discover the targets, functions, and signaling pathways involved in the pathogenesis of POP. Furthermore, the screened differentially expressed circRNAs were validated using real-time quantitative PCR (RT-qPCR), and a circRNA-miRNA-mRNA regulatory network was constructed to uncover the potential molecular mechanisms of POP pathogenesis at the transcriptional level.

## 2 Materials and methods

### 2.1 Participants

A total of 55 women who visited the West China Second University Hospital, Sichuan University, between January 2021 and July 2022, were enrolled. Of these, 28 women were diagnosed with POP, and 27 women without POP were recruited as controls. As described in a previous study (Haylen et al., 2010), the participants were diagnosed with POP according to the following criteria: POP symptoms, such as vaginal bulging, pelvic pressure, urinary dysfunction, or defecatory dysfunction; POP signs, including Stage II/III/IV evaluated by POP quantification (POP-Q) system. The exclusion criteria included malignant tumors, fibromyoma, pelvic masses, and mental abnormalities. The clinical characteristics of the study participants are presented in Table 1.

**TABLE 1:** The clinical characteristics of participants.

Characteristics	Control group (n = 27)	POP group (n = 28)	P Value
Age (years)	47.59 ± 9.37 (30–69)	61.00 ± 13.47 (26–85)	<0.0001*
BMI (kg/m^2^)	23.66 ± 2.91 (18.37–28.72)	24.00 ± 3.04 (18.73–30.04)	0.675
Gravidity	4 (2–5)	3 (2–5)	0.877
Parity	1 (1–2)	2 (1–2)	0.102
Menopause	7 (25.9%)	20 (71.4%)	0.001*
Smoking	1 (3.7%)	0 (0.0%)	0.491
Alcohol use	0 (0.0%)	0 (0.0%)	—
Hypertension	3 (11.1%)	5 (17.9%)	0.744
Diabetes	2 (7.4%)	3 (10.7%)	1.000
Hormone use	8 (29.6%)	2 (7.1%)	0.070
Estrogen use	1 (3.7%)	0 (0.0%)	0.491
Asthma	0 (0.0%)	0 (0.0%)	—
POP grade			
0/Ⅰ	27 (100.0%)	0 (0.0%)	<0.0001*
Ⅱ	0 (0.0%)	8 (28.6%)	
Ⅲ	0 (0.0%)	20 (71.4%)	
Ⅳ	0 (0.0%)	0 (0.0%)	

Data are presented as number (%), mean ± standard deviation (minimum-maximum range), or median (interquartile range). *P < 0.05 (statistically significant). BMI, body mass index.

### 2.2 Sample collection and RNA extraction

Peripheral blood samples were collected from POP and control groups. Total RNA was extracted using the TRIzol reagent (Thermo Fisher Scientific, United States) following the manufacturer’s protocol. RNA concentration and integrity were evaluated using a NanoDrop 2000 spectrophotometer (Thermo Fisher Scientific, United States) and an Agilent 2100 Bioanalyzer (Agilent Technologies).

### 2.3 CircRNA sequencing analysis

Five pairs of samples matched for age, parity, and menopausal status were selected for circRNA sequencing. Samples with an RNA Integrity Number (RIN) ≥ 7 were used for subsequent analysis. Libraries were constructed with 1 μg of RNA using TruSeq Stranded Total RNA with Ribo-Zero Gold (Illumina, United States) according to the manufacturer’s instructions, followed by sequencing with the Illumina HiSeqTM 2500 sequencing platform on 150 bp/125 bp paired-end runs.

### 2.4 Identification and quantification of human circRNA

Raw reads in fastq format were quality filtered using Trimmomatic software (Bolger et al., 2014) and clean reads were obtained. The clean reads were then mapped to the human genome (GRCh38) using the HISAT2 software (Kim et al., 2015). The CircRNA Identifier program (v2.0.3) was used to identify and characterize the circRNAs and the circRNAs obtained were aligned with the CircBase, CircAtlas, and CIRCpedia databases to identify known and predicted circRNAs. The spliced reads per million mapping (RPM) algorithm was employed to calculate the expression of circRNAs. DESeq software (v1.18.0) was used to screen DEcircRNAs based on the criteria of fold change >2.0, and P < 0.05.

### 2.5 RT-qPCR validation

Specific divergent primers of circRNAs were designed using CircPrimer 2.0 and Primer3 software. The divergent primer sequences are shown in Supplementary Table S1.

To quantify the amount of circRNAs, cDNA was synthesized using the PrimeScript™ RT reagent Kit with gDNA Eraser (TaKaRa, China) using 1 μg of total RNA. RT-qPCR analyses were performed using QuantiNova™ SYBR^®^ Green PCR Kit (Qiagen, Germany) and a Roche cobas^®^ z480 real-time PCR system (Roche Molecular System Inc., United States). The quantitative PCR products of the circRNAs were confirmed by Sanger sequencing. The β-actin was employed as an internal control. The relative expression of circRNAs was calculated by the 2^−ΔΔCT^ method.

### 2.6 Bioinformatic analysis

Gene Ontology (GO) and Kyoto Encyclopedia of Genes and Genomes (KEGG) enrichment analyses were performed to predict the potential biological functions of dysregulated circRNAs. Target miRNAs of DEcircRNAs and downstream target genes were predicted using miRanda (v3.3a) software to obtain circRNA-mRNA pairs. Moreover, circRNA-mRNA co-expression analysis was performed and positively correlated circRNA-mRNA pairs were screened. Based on overlapping circRNA-mRNA pairs, the ceRNA network of circRNA-miRNA-mRNA was constructed.

### 2.7 Statistical analysis

Statistical analyses were performed using the GraphPad Prism (v8.0). Comparisons between groups were performed using an unpaired t-test. Data are presented as mean ± standard deviation. P value <0.05 was considered statistically significant. The diagnostic value of these DEcircRNAs was evaluated using ROC curves.

## 3 Results

### 3.1 DEcircRNAs in human blood

**FIGURE 1:**
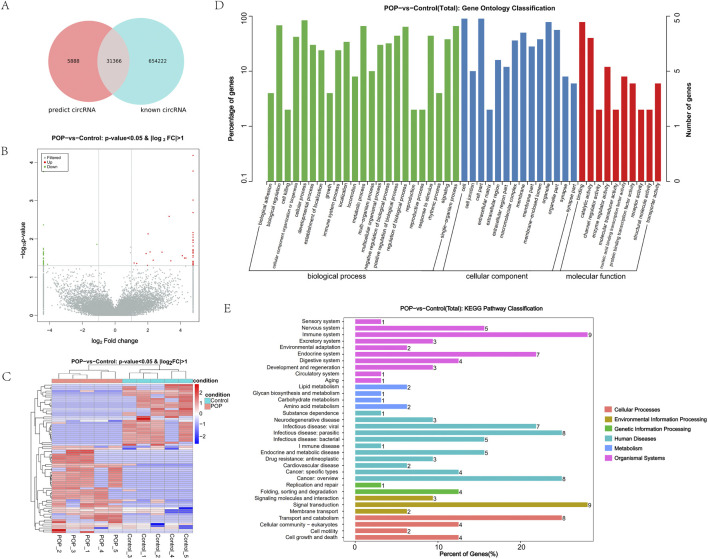
Identification of DEcircRNAs in human blood for POP and GO analysis and KEGG pathway annotation of DEcircRNAs. **(A)** The Venn diagram shows the circRNAs predicted with circRNA sequencing analysis and known circRNAs contained in the circBase, which are illustrated as red circle and blue circle, respectively. The circRNAs predicted include overlapping known circRNAs and non-overlapping novel circRNAs. **(B)** The volcano plot presents DEcircRNAs screened with the criteria of fold change >2.0 and P < 0.05. Upregulated and downregulated circRNAs are illustrated as red dots and green dots, respectively. **(C)** The heat map shows the DEcircRNAs between POP group and control group. **(D)** The terms of biological process, cellular component, and molecular function related to DEcircRNAs. **(E)** The most significant pathways that might activate POP pathogenesis.

In total, 37,254 circRNAs were detected. Among them, 31,366 circRNAs were found in circBase and 5,888 novel circRNAs were identified, which are presented in a Venn diagram (Figure 1A). 75 DEcircRNAs were selected based on fold change and P values (fold change >2.0, P < 0.05), as shown in the volcano plot (Figure 1B). The heat map indicated that DEcircRNAs were divided into two clusters, suggesting intergroup differences and intragroup consistency between the POP and control groups (Figure 1C). Among the screened 75 DEcircRNAs, 44 were upregulated and 31 were downregulated.

### 3.2 Functional analysis of DEcircRNAs

GO and KEGG enrichment analyses were performed on the 75 DEcircRNAs according to their host genes to investigate their potential biological functions. According to the GO enrichment results, 346 biological process terms, 95 cellular component terms, and 117 molecular function terms were significantly enriched for the DEcircRNAs. The main biological processes involved in POP were natural killer (NK) cell proliferation, NK T cell proliferation, and positive regulation of cytolysis. Cellular component analysis suggested that asymmetric synapses, symmetric synapses, and myelin sheath abaxonal regions were highly activated in POP group. The most enriched molecular function terms were 1-phosphatidylinositol-3-kinase regulator, histone methyltransferase, and polynucleotide 5′-hydroxyl-kinase activities (Figure 1D).

KEGG pathway analysis showed that the host genes of the DEcircRNAs were mainly enriched in signal transduction, the immune system, transport, and catabolism (Figure 1E).

### 3.3 Validation of DEcircRNAs

**FIGURE 2:**
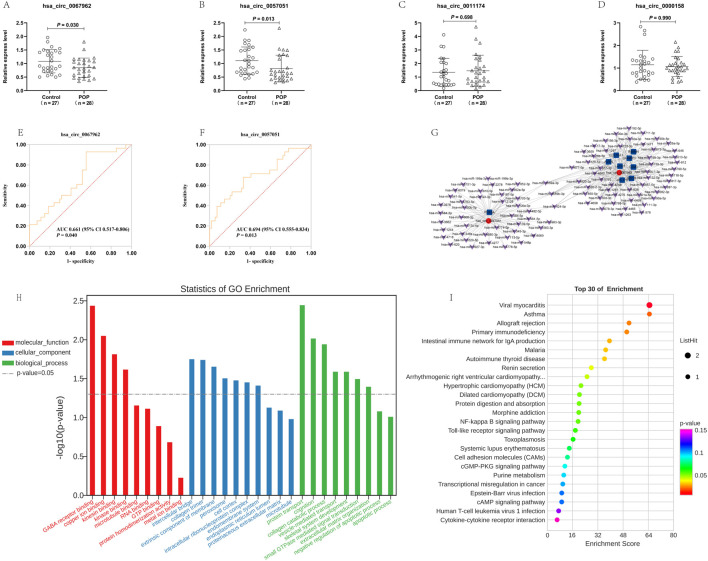
Relative expression of selected DEcircRNAs, including **(A)** hsa_circ_0067962, **(B)** hsa_circ_0057051, **(C)** hsa_circ_0011174, and **(D)** hsa_circ_0000158 in blood from women with POP and controls detected by RT-qPCR. ROC curve analysis for POP women based on **(E)** hsa_circ_0067962, and **(F)** hsa_circ_0057051 expression. **(G)** CeRNA analysis of circRNA-miRNA-mRNA. The circRNAs, miRNAs, and mRNAs are labeled as red circles, purple arrows, and blue squares, respectively. **(H)** GO terms enriched in the mRNAs regulated by two validated DEcircRNAs. **(I)** The top 30 KEGG pathways enriched in the mRNAs regulated by two validated DEcircRNAs.

Three upregulated and one downregulated circRNAs (Supplementary Table S1) were selected based on their highest expression, fold change >2.0, and P < 0.05, for validation by RT-qPCR. The results revealed that the expression levels of two circRNAs (hsa_circ_0067962 and hsa_circ_0057051) were significantly lower in the blood of the POP group than those in the blood of the control group, whereas the expression levels of the other two circRNAs were not significantly different (Figures 2A–D). Furthermore, ROC curve analysis was performed to determine the diagnostic value of the two validated DEcircRNAs in POP. The areas under the ROC curve (AUC) for hsa_circ_0067962 and hsa_circ_0057051 were 0.661 (95% CI 0.517–0.806; P = 0.040) and 0.694 (95% CI 0.555–0.834; P = 0.013), respectively (Figures 2E, F; Supplementary Table S2).

### 3.4 CeRNA analysis of validated DEcircRNAs

CeRNA network analysis was conducted for the two validated DEcircRNAs (hsa_circ_0067962 and hsa_circ_0057051). A total of 301 circRNA-miRNA interaction relationships were identified, and the top 100 circRNA-miRNA pairs are illustrated (Supplementary Figure S1). Furthermore, 96 circRNA-mRNA pairs were obtained from miRanda prediction, and 54 circRNA-mRNA pairs were screened based on co-expression analysis. Nine overlapping circRNA-mRNA pairs (hsa_circ_0067962/COL10A1, hsa_circ_0067962/LACC1, hsa_circ_0067962/RBM44, hsa_circ_0067962/AVEN, hsa_circ_0067962/JAKMIP1, hsa_circ_0057051/PDE3A, hsa_circ_0067962/SGCB, hsa_circ_0067962/ARL17B, and hsa_circ_0067962/CD40) were used, together with 93 common targeting miRNAs, to obtain a circRNA-miRNA-mRNA network (Figure 2G).

GO and KEGG pathway analyses were performed for the two validated DEcircRNAs according to their ceRNA pairs. The most enriched biological process terms were protein transport, cognition, and collagen catabolism. The most enriched cellular component terms were intercellular bridge, collagen trimer, and extrinsic component of the membrane. The most enriched molecular function terms were the gamma-aminobutyric acid (GABA) receptor, copper ion, and kinesin binding (Figure 2H). The pathways were mainly enriched for viral myocarditis, asthma, and allograft rejection (Figure 2I).

## 4 Discussion

Pelvic organ prolapse is a prevalent clinical diagnosis with deleterious effects on women’s health. Numerous studies have been conducted to detect gene expression profiles in pelvic tissue, including the vaginal wall, uterosacral and round ligaments, and pubococcygeus muscle, from women with POP and control women (Yu et al., 2022). However, the underlying molecular mechanisms remain unclear. Many studies have revealed that circRNAs are implicated in multiple diseases. However, the roles of circRNAs in POP remain largely unknown.

Here, we investigated the expression profiles of circRNAs in the peripheral blood of POP and control groups. A total of 75 DEcircRNAs were identified. These DEcircRNAs were enriched in biological processes of NK cell proliferation, and NK T cell proliferation. The related KEGG pathways included immune system. The infiltration level of NK T cell in POP group is higher than that of the non-POP group (Wu et al., 2023). Three differential immune cell types were identified between POP and non-POP tissues (Zhao et al., 2020). These results suggested that DEcircRNAs may be involved in POP pathogenesis through immune cells.

The results of RT-qPCR showed that hsa_circ_0067962 and hsa_circ_0057051 were significantly downregulated in women with POP. A circRNA-miRNA-mRNA regulatory network was constructed for the two DEcircRNAs. A total of 93 miRNAs were identified. Of these, four miRNAs (hsa-miR-638, has-miR-620, hsa-miR-139-5p and has-miR-612) may be involved in muscle tissue regulation. The hsa-miR-638 was downregulated in the skeletal muscle of patients with amyotrophic lateral sclerosis (Aksu-Menges et al., 2022). The hsa-miR-620 increased proliferation of airway smooth muscle cells (Chen et al., 2020). The hsa-miR-139-5p was downregulated in hypertrophic cardiomyopathy (Sun et al., 2021). The has-miR-612 was involved in cardiomyocyte proliferation. 52 miRNAs have been reported to play regulatory roles in several diseases. However, the roles of these miRNAs in POP remain unclear. The remaining 37 miRNAs have not been previously reported.

The following nine mRNAs were included in the ceRNA network: COL10A1, LACC1, RBM44, AVEN, JAKMIP1, PDE3A, SGCB, ARL17B, and CD40. COL10A1 is a secreted collagen. COL10A1 expression was positively correlated with estrogen receptor (ER) status, while ER-α levels in the uterine ligaments of women with POP were lower than those in the control group (Lang et al., 2003; Zhang et al., 2020), suggesting that COL10A1 expression may be downregulated in women with POP. Based on the ceRNA pairs of hsa_circ_0067962/COL10A1, COL10A1 was presumed to be downregulated in the POP group, which is consistent with the literature search results. These results suggested that COL10A1 may be involved in POP through the ER. LACC1 interacted with nitric oxide synthase to exert anti-inflammatory effect. A study showed that upregulation of nitric oxide synthase may cause bladder muscle relaxation (Ho et al., 2004). LACC1 may play an anti-inflammatory role in pelvic floor disorders via nitric oxide synthase.

RBM44 is a germ cell intercellular bridge protein. RBM4 limited IL-6 protein synthesis to exert anti-inflammatory effect (McClure et al., 2015), and IL6 potentially play an important role in POP progression (Zhou et al., 2018), suggesting that RBM44 may be involved in POP development by regulating inflammatory response. AVEN is an antiapoptotic protein that blocks pathological apoptosis by stabilizing Bcl-xL (Kutuk et al., 2010). The expression level of Bcl-xL was significantly higher in the POP group than in the non-POP group (Saatli et al., 2014). AVEN may exert an anti-apoptotic effect on POP by binding to Bcl-xL.

JAKMIP1 is a critical regulator of mRNA translation in the mammalian brain (Berg et al., 2015). JAKMIP1 expression was positively correlated with β-catenin accumulation (Okai t al., 2013), and β-catenin may be downregulated in POP (Gong et al., 2021), suggesting the lower expression of JAKMIP1 in POP group. In the ceRNA pairs of hsa_circ_0067962/JAKMIP1, JAKMIP1 was presumed to be downregulated in the POP group. JAKMIP1 may participate in POP through β-catenin signaling pathway. PDE3 regulated AMPK in various cell types (Dillard et al., 2020), and AMPK signaling pathway could activate stem cells within the pelvic floor muscles (Kang et al., 2019), suggesting that PDE3A may be associated with pelvic floor muscle cell activation. Mutations in the SGCB gene resulted in limb-girdle muscular dystrophies, characterized by pelvic muscle weakness and wasting (Magri et al., 2022), which may be involved in the pathogenesis of POP.

ARL17B is a multiple sclerosis-associated gene. The relationship between ARL17B and POP remains unclear. The expression of plasma CD40L is increased in pelvic inflammatory diseases, however, the relationship between CD40 and POP is unclear. Here, the regulatory mechanism of the circRNA-miRNA-mRNA network in POP remains unclear. However, we provide ideas for future research on POP-related circRNAs and their regulatory mechanisms.

GO and KEGG enrichment analyses were performed for hsa_circ_0067962 and hsa_circ_0057051. The GO term was enriched in the collagen catabolic process. Collagen is an important component of the extracellular matrix in connective tissues and maintains supportive functions in the pelvic floor. Abnormalities in collagen catabolism could destroy the supportive function of the pelvic floor and were associated with POP development (Gong and Xia, 2019).

Here, circRNA sequencing was used to investigate the circRNA expression profiles in POP. Single-cell RNA sequencing was reported to be used to construct a transcriptomic atlas of individual cells in the vaginal wall of POP and control groups, and extracellular matrix dysregulation and immune reactions involvement were detected (Li et al., 2021; Miao et al., 2023), which is consistent with our findings. In future studies, we will include larger cohorts to confirm these circRNA results and include age as a control factor. In addition, we will integrate the peripheral blood omics data and tissue omics data of POP to further explain the pathogenesis of POP.

## 5 Conclusion

This study provides comprehensive circRNA expression profiles in the peripheral blood of POP and control groups. The DEcircRNAs were enriched in NK cell proliferation biological process and immune system pathway, suggesting that immune inflammation may be involved in POP. The hsa_circ_0067962 and hsa_circ_0057051 were significantly downregulated in the POP group, which mainly involved in the collagen catabolic process. Furthermore, we constructed a circRNA-miRNA-mRNA network that may provide new insights into POP pathogenesis.

## Data Availability

The datasets presented in this study can be found in online repositories. The names of the repository/repositories and accession number(s) can be found below: https://ngdc.cncb.ac.cn/gsa-human, HRA009240.
