# Supplementary material for: Comprehensive analysis of the circRNA expression profile and circRNA-miRNA-mRNA network in pelvic organ prolapse
Source: Front Genet. 2025 Jan 20;15:1527223. doi: 10.3389/fgene.2024.1527223 (PMC11788335; doi:10.3389/fgene.2024.1527223)
Supplement: Supplementary file 1 [file DataSheet1.docx]

Supplementary Table S1 Divergent primers of circRNAs for RT-qPCR.

| circBase ID | Forward primer | Reverse primer |
| --- | --- | --- |
| hsa_circ_0067962 | CAGTTTGCCCCGTTCATCTG | CCCGCGTGTCCAGATTTTC |
| hsa_circ_0057051 | CCAACAGGGAGAACCATAAAGGAG | CCCAGCTCAAGTATCTGAATTTGC |
| hsa_circ_0011174 | CACTCACCCTTCCGAACTCT | TCCGGCCACTGTATCGAAAT |
| hsa_circ_0000158 | GCTGCTCTGAAGGAACAAACTTA | CTCCAAAGGCTCACACTGAATC |
| β-actin | CATGTACGTTGCTATCCAGGC | CTCCTTAATGTCACGCACGAT |

Supplementary Table S2 Diagnostic value of validated DEcircRNAs for POP.

| Parameter | hsa_circ_0067962 | hsa_circ_0057051 |
| --- | --- | --- |
| AUC | 0.661 | 0.694 |
| Sensitivity (%) | 57.1 | 67.9 |
| Specificity (%) | 59.3 | 66.7 |
| Positive predictive value (%) | 59.3 | 67.9 |
| Negative predictive value (%) | 57.1 | 66.7 |


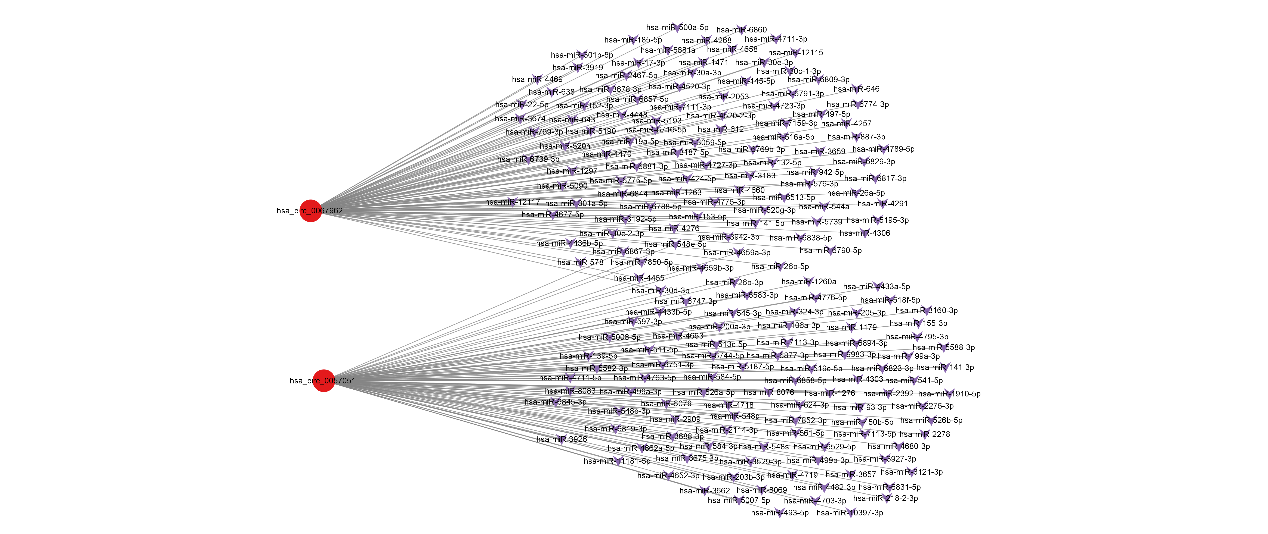


**Supplementary Figure** S**1**

The top 100 circRNA-miRNA interaction network. The circRNAs are labeled as red circles, and miRNAs are labeled as purple arrows.
